# Redesigning healthcare: The 2.4 billion euro question? Connecting smart technology to improve outcome of patients

**DOI:** 10.1007/s12471-016-0834-6

**Published:** 2016-04-06

**Authors:** R. W. Treskes, E. T. Van Der Velde, D. E. Atsma, M. J. Schalij

**Affiliations:** Department of Cardiology, Leiden University Medical Center, Leiden, the Netherlands

**Keywords:** e-Health, Electronic health records, Healthcare costs

## Abstract

Although it has been possible to transfer electrocardiograms via a phone line for more than 100 years, use of internet-based patient monitoring and communication systems in daily care is uncommon. Despite the introduction of numerous health-monitoring devices, and despite most patients having internet access, the implementation of individualised healthcare services is still limited. On the other hand, hospitals have invested heavily in massive information systems offering limited value for money and connectivity. However, the consumer market for personal healthcare devices is developing rapidly and with the current healthcare-related investments by tech companies it can be expected that the way healthcare is provided will change dramatically. Although a variety of initiatives under the banner of ‘e-Health’ are deployed, most are characterised by either industry-driven developments without proven clinical effectiveness or individual initiatives lacking the embedding within the traditional organisations. However, the introduction of numerous smart devices and internet-based technologies facilitates the fundamental redesign of healthcare based on the principle of achieving the best possible care for the individual patient at the lowest possible cost.

*Conclusion* The way healthcare is delivered will change, but to what degree healthcare professionals together with patients will be able to redesign healthcare in a structured manner is still a question.

## Introduction

Approximately 110 years ago, Willem Einthoven was the first person to use telemedicine by sending clinically obtained ECGs by telephone to his laboratory located outside the hospital, because his ECG machine was not allowed on the wards [1]. It took another 80 years, however, to invent and distribute the personal computer (PC, introduced 1981). This event marked the beginning of the widespread use of PCs [2]. In 1991, the ‘World Wide Web’ or internet was introduced, thereby allowing computers to exchange digital information, and within 5 years companies in different sectors started offering services via internet [3]. By 2013, 97 % of the Dutch population had internet access and online banking and online shopping were used by 83 and 82 %, respectively, of this group [4]. Furthermore, in the Netherlands 90–98 % of all inhabitants can use fourth-generation mobile networks (allowing mobile access to fast internet) and 85–95 % of the land area is covered by the two dominant mobile providers.

Computers have also revolutionised healthcare. Laboratory results, diagnostic images and patient records became available online in most hospitals and it became possible to exchange data between healthcare providers. General practitioners (GP) also use PCs to record patient data, and currently 98 % of Dutch GPs are storing information in electronic health records.

The current hospital information systems (HIS), however, still have major limitations. Systems are expensive, complex, and connectivity of most systems is limited. Furthermore, commercially developed/used applications such as video-consultation systems, telediagnosis and teletreatment systems are only used by a small number of healthcare providers. The possibilities to extract data out of these applications into the HIS are still limited [5]. With respect to the costs of the commercially available HIS, it is remarkable that hospitals are still willing to invest enormous amounts of money and human resources in generic mainstream systems, offering limited value on investment whereas it can be expected that in the near future information will be stored in the cloud and networked distributed applications will provide optimal support for treating individual patients. A simple calculation of costs returns an astonishing 2.4 billion euros spent by approximately 80 Dutch hospitals to introduce a basic HIS [6]. Maintenance and regular updates of these complex systems are consuming even more money and require large numbers of staff. Money not spent on direct care and presumably to be written off in the near future, as will be discussed.

In the meantime, the consumer market for personal health-monitoring devices and systems is developing rapidly. At present, patients frequently provide their doctors with data obtained using these devices. Although in line with the wish to provide patients with tools to monitor their health, it is not possible to incorporate data obtained with these devices into the HIS to obtain a comprehensive and patient-specific dataset. However, with the current healthcare-related investments by companies such as Google, Apple, Microsoft, Samsung and Philips it can be expected that the way healthcare is provided will change in the near future. Patients, used to 24/7 services provided by airlines, banks and travel agencies, will demand similar individualised healthcare services. Living in an era in which one can book a flight to a place anywhere in the world at any time, patients will no longer accept waiting weeks for an appointment with a doctor and not getting feedback within a few hours in case of questions. Although a variety of initiatives are currently deployed, most are characterised by either industry-driven developments without proven clinical effectiveness or individual initiatives lacking the embedding within the traditional organisations. Furthermore, most of these initiatives, under the trendy banner of ‘e-Health’, lack any fundamental thought about the way healthcare should develop. Additionally, the entity e‑Health is ill defined and may vary from simple email-based patient-physician conversations to complex diagnostic systems continuously monitoring the health of patients. Last but not least, e‑Health is of no value if it is not part of a larger, preconceived plan to improve healthcare. In other words, it is more a question of fundamentally redesigning healthcare with the help/aid of new technologies than of the introduction of healthcare-related gadgets per se without defining how to improve the quality of the provided care.

In this paper we will try to outline the future of healthcare by discussing a patient’s journey.

## Patient’s journey

**Fig. 1:**
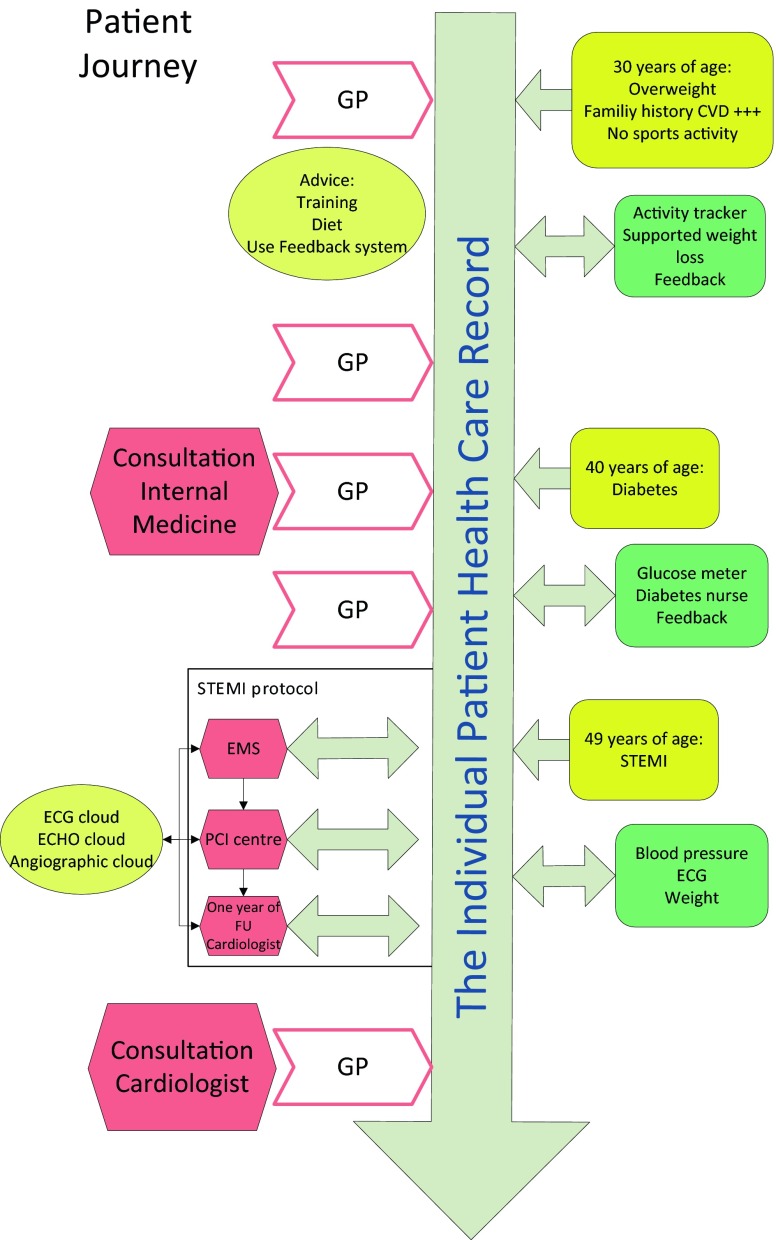
Patient’s journey.

Let’s assume a 30-year-old male patient with no relevant medical history except for a positive family history of cardiovascular disease (Fig. 1). The patient is overweight and not performing any physical exercise. Without intervention, this patient is at increased risk of developing cardiovascular disease before the age of 60. The patient visits his GP because of some minor illness.

So how to start? First we have to inform the patient about his risk profile, involve him in a training program and educate him. To achieve this, we activate his personal healthcare record (PHCR) and provide him with educational materials. The patient will buy an activity tracker connected to a secure cloud, giving him feedback about his accomplishments in comparison with age-matched peers. Data from the activity tracker will be stored in the PHCR. Furthermore, the patient will receive advice on how to proceed. Every year he visits his GP who can retrieve data from the PCHR and provide feedback to the patient. So far so good.

At the age of 40, the patient develops diabetes. His GP consults the internal medicine specialist and gives the patient personal diabetes advice. Furthermore, the patient receives a Bluetooth glucose meter. Data from this meter are sent to PCHR and monitored by the GP.

Despite all efforts, the patient has a myocardial infarction at age 49. He activates the emergency service who connects to his PCHR to evaluate history, current medication and known allergies. Upon arrival, the ambulance crew establishes the diagnosis at his home, and transfers him to the nearest percutaneous coronary intervention (PCI) centre. After the PCI procedure, the patient is treated according to the guidelines, provided with Bluetooth devices enabling him to send data about his heart rhythm, blood pressure and weight to his PCHR and is soon seen at the video consultation clinic. In the next year, regular outpatient consultations are alternated with video consultations and after one year he is referred to the GP who has secure access to all relevant data from the cardiology record. During the following years, the GP electronically asks for advice from the cardiology clinic on a regular basis and the patient is doing fine.

Is this a futuristic impression or just regular care waiting to be implemented? All the different devices and the necessary technical infrastructure are already available; however, borders between different healthcare segments are preventing a widespread implementation.

For this to become reality, training of healthcare providers should change, patients have to be involved in redesigning the system and reimbursement systems should be able to finance healthcare chains rather than individual actors. Furthermore, instead of focussing on too-big-to-fail HIS all efforts should be focused on developing dedicated applications connected to each other to allow the best individualised care at the lowest possible costs.

## e-Health

As stated above, e‑Health is not very well defined [7, 8]. The Dutch Board of Public Health (Raad voor Volksgezondheid, RVZ) defines e‑Health as ‘the use of new information and communication technologies, especially internet technology, to support and improve health and healthcare’ [9]. The World Health Organisation (WHO) on the other hand defines e‑Health as ‘the transfer of health resources and healthcare by electronic means’ [10]. This difference is significant, as the RVZ’s definition indicates a supportive approach to e‑Health, whereas the WHO’s definition indicates a more substituting approach. E‑health, irrespective of the definition used, remains a broad term. To clarify this we propose to subdivide e‑Health into separate entities as shown in Table 1.

**Table 1:** Different entities of e‑Health

E-public health	Encompasses all actions taken using information technology to improve and protect health on a society level
E-support	Encompasses logistical actions needed in healthcare, such as patient access to their own patient files/medical records
E-care	Supports the interview, physical examination, treatment and follow-up using electronic devices
Telemonitoring	The process in which patient parameters are measured remotely. Various devices measuring for instance blood pressure, electrical activity of myocardial cells, oxygen saturation and patients weight are used in clinical practice [17]
Teletreatment	The process in which patients are treated from a remote distance [17]
Teleconsultation	The process in which doctors are consulted from a remote location, using video or email technology, by either colleagues or patients [17]
Telediagnosis	The determination of the nature of a disease at a site remote from the patients on the basis of telehealth methods of transmitted data [17]

## Impact of e‑Health implementation

There are numerous examples of e‑Health-related studies in the literature. The problem in comparing them is that, although two studies can both be e‑Health-related, the methods used can differ substantially. Diseases subject to numerous e‑Health research projects are arterial hypertension, diabetes and heart failure (HF). Several studies have demonstrated positive effects of telemedicine on outcome of both arterial hypertension and diabetes patients [11].

The results of telemedicine on the outcome of HF patients are, however, conflicting. Telemonitoring studies using implantable cardioverter-defibrillators (ICDs) demonstrated that remote monitoring of the ICD in HF patients enhances life expectancy and reduces the number of related clinical events [12]. The value of phone support systems on the other hand remains debatable. One systematic review found that phone support systems reduced hospitalisation and all-cause mortality in HF patients [13]. However, a large clinical trial comprising 826 HF patients randomised to a phone-based telemonitoring system, and 827 HF patients randomised to regular care found no differences in all-cause mortality or hospital readmission rates. Moreover, there were no differences in the number of patients readmitted for HF, the number of days in hospital or number of hospitalisations [14].

## Where to go from here?

With the introduction of all kinds of smart devices and internet-based technologies, it is possible to redesign healthcare. What do we need? First of all, define the needs of the individual patients, so involve them in the design process. Secondly, introduce dedicated applications to provide both patients and healthcare providers with the optimal information needed at the correct time and place. These applications should be connected to the patient data stored safely in cloud-based systems. Data stored in these systems should be available for registration purposes, to get reimbursement and to benchmark healthcare systems.

The leading principle as stated by Porter in 2012 should be: ‘achieving high value for patients must become the overarching goal of healthcare delivery, with value defined as the health outcomes achieved per dollar spent’ [15].

The present generic HIS lack all the criteria defined above. Firstly, initial costs (30–60 million euro per hospital) and costs to keep systems up-to-date are extreme. Secondly, due to the top-down generic design principles it is difficult to fulfil all the wishes from the different healthcare providers for all scenarios encountered and most systems are full of compromises. Furthermore, due to the obsession of all the parties involved (insurance companies, financing departments, inspectorates, ministry of health, scientific societies) to register, store and report an ever-growing dataset that can be accessed always and everywhere, the design of currently used HIS may lead to the so-called information completeness paradox. In managerial cultures, completeness of data is thought to be a kind of holy grail helping to be in control. However the overload of accumulated data with no particular hierarchy in a patient file may, especially in critical situations, prevent the healthcare professional from taking the correct decisions [16]. Furthermore the current systems are, despite some initiatives, not really informative for the patients themselves.

**Fig. 2:**
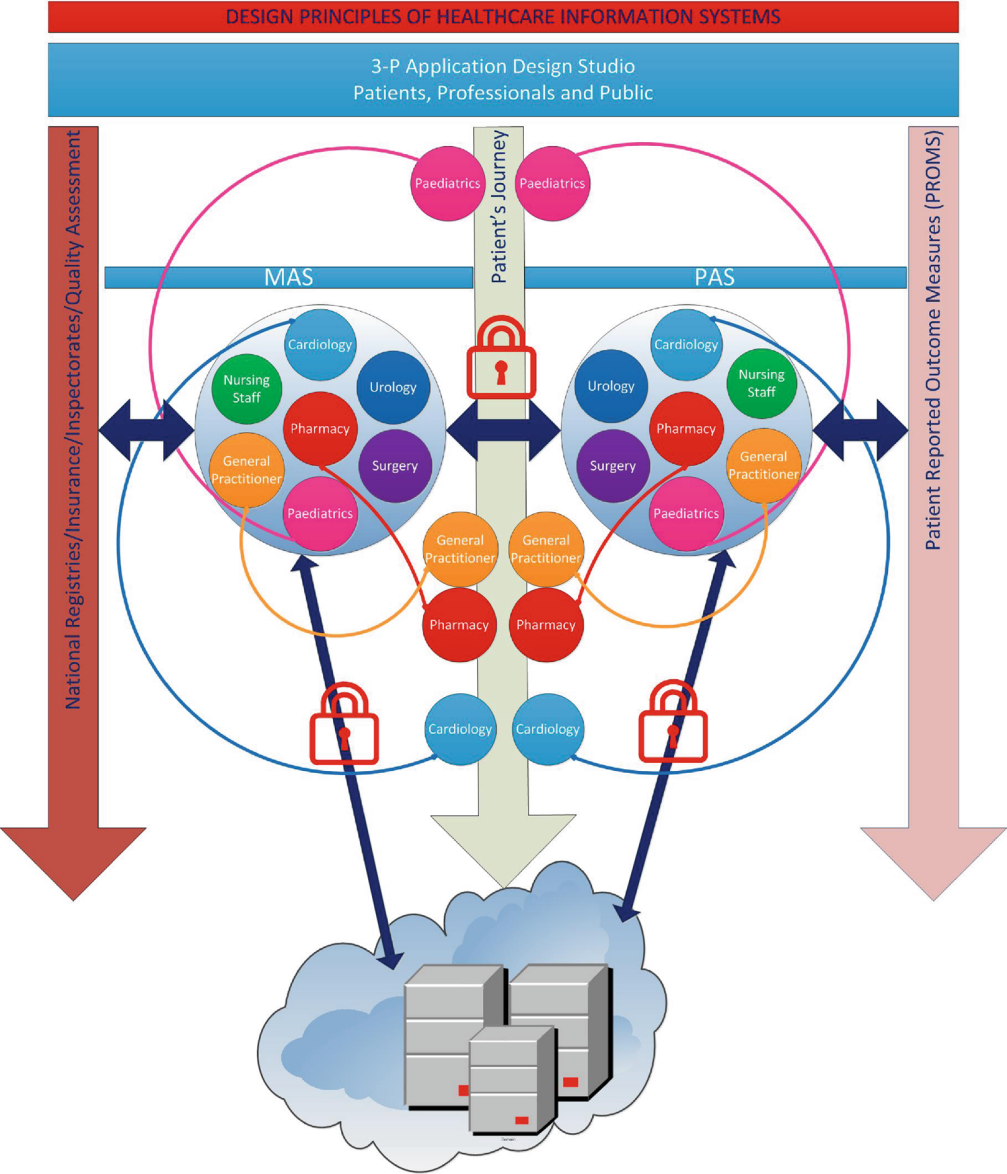
Design principles of healthcare information systems

Therefore, in order to regain control it should be realised that information systems in themselves add no value, but that the value comes from how information is handled. So how to start? By adopting a leading design principle based on the SET (Safe, Effective and Transparent) principles. How to translate this into practice? It is envisioned that a patient has his own patient records stored in the cloud. This virtual record starts at birth and continues to build up during life. In this virtual record all healthcare-related events are stored. Every healthcare provider involved works with a dedicated application which can be obtained from a Medical Application Store (MAS). The MAS contains specific certified applications in which information is gathered and stored in the patient virtual healthcare record. Each stakeholder in the medical field has his own application: cardiology, pulmonology, pharmacy, general practitioners, nursing staff, etc. If a patient is admitted to the department of a relevant stakeholder, that stakeholder activates his or her application and stores the information. In the example (Fig. 2), the paediatrician switches on the paediatrics application at age 5. At the very same time, the information is automatically translated into lay terms, making it understandable for patients. This information is visible in a patient-specific analogue of the application, obtained by the patient from the Patients Application Store (PAS). Each application in the MAS, has an equivalent in the PAS. The PAS contains patient specific information, which encompasses a lay description of the data gathered in the MAS (and made available in the patient cloud), as well as both general and specific information, such as instruction videos and the anatomy and physiology of a relevant organ. The MAS furthermore contains a separate folder in which the physician can write notes which will not be copied to the PAS. The content of MAS and PAS is defined by relevant stakeholders, including medical specialists, patient organisations and educational specialists. Applications should be based on open source software. Furthermore, connectivity and data exchange should be easy. As suggested in Fig. 2, content, design, and certification are brought together in the so-called 3‑P application design studio (Patient, Professionals and Public). Following this software structure, the information will be to-the-point to the physician, understandable to the patient and transparent to the public. Structured storage of information enables easier data extraction for quality assessment purposes [18].

## e-Health implementation

Despite the potential advantages of redesigning healthcare as discussed in the previous section several problems may hamper the rapid and widespread implementation of e‑Health. First, instead of reimbursing individual healthcare providers, thereby creating artificial borders between the different sectors, it will be necessary to reimburse healthcare systems. Currently, however, several financial constraints for e‑Health implementation are repeatedly reported and summarised in Table 2. Second, it is important to recognise possible hesitations by the involved stakeholders as summarised in Table 3. Third, it is important that data safety is ensured and monitored by an independent inspectorate. Furthermore it is vital that patients are able to refuse the exchange of their data between healthcare providers and that access rights are well described.

**Table 2:** Reimbursement

What is reimbursed?	Problems with reimbursement?
Screen-to-screen contacts	Teleconsultations from a general practitioner to a medical specialist are not reimbursed (currently, only teledermatology is reimbursed)
Telemonitoring (after negotiation with healthcare insurance companies)	Reimbursement is not in proportion to the time an e‑Health intervention takes
Telemonitoring and screen-to-screen contacts (STSC) in which the patient is contacted using video-telephony, can be reimbursed	Reimbursed only if a STSC is both a substitute for a face-to-face outpatient clinic visit and if this STSC is a follow-up visit of a previous face-to-face outpatient clinic visit

**Table 3:** Mindset of involved stakeholders

Medical staff	1. Are overwhelmed by information from electronic health records and devices 2. Experience a lack of reimbursement for e‑Health 3. Have concerns about the quality of the data generated by e‑Health and m‑Health devices
IT specialists	Are not well enough instructed on what health information doctors need at what time
Patients	1. Do not always understand what is written down in their electronic health record, because of what is often referred to as ‘doctors language’ 2. Sometimes lack proper experience with information technology, especially smartphone technology
Managers	Have concerns about the logistics of control of the data
Nursing staff	Are afraid that they will be overwhelmed with data

It will be important to address these barriers in redesigning healthcare and to stimulate all involved. In other words, change the mindset!

## Conclusions

The question is not if the way we provide healthcare will change, but to what extent healthcare professionals together with patients will be able to fundamentally redesign healthcare in a structured manner. This process should start with defining the needs of patients based on the principle of best achievable care at the lowest possible costs.
